# Optically Induced Thermal Gradients for Protein Characterization in Nanolitre-scale Samples in Microfluidic Devices

**DOI:** 10.1038/srep02130

**Published:** 2013-07-04

**Authors:** D. M. Sagar, Samir Aoudjane, Matthieu Gaudet, Gabriel Aeppli, Paul A. Dalby

**Affiliations:** 1 Department of Biochemical Engineering, Torrington Place, University College London, London, WC1E 7JE, U.K; 2 London Centre for Nanotechnology and Department of Physics and Astronomy, University College London, London, WC1H 0AH, U.K; 3 Division of Infection & Immunity, UCL, Cruciform Building, 90 Gower Street, LONDON WC1E 6BT

## Abstract

Proteins are the most vital biological functional units in every living cell. Measurement of protein stability is central to understanding their structure, function and role in diseases. While proteins are also sought as therapeutic agents, they can cause diseases by misfolding and aggregation in vivo. Here we demonstrate a novel method to measure protein stability and denaturation kinetics, on unprecedented timescales, through optically-induced heating of nanolitre samples in microfluidic capillaries. We obtain protein denaturation kinetics as a function of temperature, and accurate thermodynamic stability data, from a snapshot experiment on a single sample. We also report the first experimental characterization of optical heating in controlled microcapillary flow, verified by computational fluid dynamics modelling. Our results demonstrate that we now have the engineering science in hand to design integrated all-optical microfluidic chips for a diverse range of applications including in-vitro DNA amplification, healthcare diagnostics, and flow chemistry.

Protein stability measurement is key to the fundamental understanding of protein structure, folding and function in biology, as well as their role in diseases that result from misfolding and aggregation. The desire to characterise all human proteins and identify those that lead to diseases such as Alzheimer's and Parkinson's, as well as novel drugs that target them, is driving the need for low-volume and high throughput assays that can accurately measure protein stability, ligand binding affinity and kinetics of protein unfolding or aggregation^1^,^2^,^3^,^4^,^5^,^6^,^7^.

We recently established a microfluidic approach to measure the extent of protein unfolding by pre-equilibrating a series of nanolitre samples with chemical denaturants, and measuring their intrinsic protein fluorescence^8^. This gave equilibrium stability measurements of the protein FKBP-12, and also the binding affinity of FKBP-12 to a small molecule drug compound, demonstrating the potential for drug discovery. We also used the technique to determine the impact of a mutation upon FKBP-12 stability, illustrating the power of the method for analysing the role of natural protein variations in genetic disorders. However, the use of chemical denaturants limited the throughput of protein stability measurements in nanolitre samples, as each stability curve requires typically 20–25 samples at different denaturant concentrations, and sample pre-equilibration for at least one hour.

Here we combine a number of factors synergistically to significantly improve upon this throughput for protein stability, while also increasing the functionality to include kinetic measurements of protein denaturation. Thermal denaturation in classical larger-scale experiments improves throughput by step-wise ramping the temperature of a sample while continuously taking measurements^1^,^2^,^9^,^10^,^11^,^12^,^13^,^14^. This also avoids the need to introduce additional chemical denaturants into the sample. Thermal unfolding is also typically faster than chemical denaturation^8^,^15^,^16^,^17^, which therefore shortens the time to reach equilibrium. Microfluidics can minimise the sample volume and material required, but crucially also allow more rapid equilibration of the fluid temperature to that of the surrounding materials. Finally, optical stimulation provides direct sample heating for instantaneous temperature ramping and thermal control^18^. By contrast, the commonly used Peltier or resistive elements heat samples indirectly by conducting heat through the container walls. Such heating elements themselves take time to reach thermal equilibrium, and require electrical connections. While small but rapid temperature jumps have been induced in mL sample volumes using high-powered IR lasers for many years^18^,^19^,^20^, the matching of microfluidic sample volumes to IR laser spot dimensions enables much greater temperature excursions as demonstrated previously for samples between glass plates or in microfluidic chambers^18^,^19^.

IR-induced heating has been successfully applied to PCR for static samples^18^, in microcapillaries placed on a Si wafer cooled by Peltier elements. The work described here is different in that we are probing protein stability in a two-laser experiment, using a plug-and-play optical fiber connected to a device mountable on a microscope stage. This has allowed us to obtain the first high-resolution three-dimensional images of stable thermal gradients in a microcapillary with point source IR heating. The technique allows any desired temperature profile to be achieved by a combination of controlled mass-flow and optically-induced heating. Thermal gradients radiating away from the point source permit temperature-dependent protein denaturation curves to be imaged in space, and over the ms-mins timescales relevant to biological function, protein unfolding or aggregation. As a demonstrator, we measured the kinetics of unfolding of the 238 residue green fluorescent protein (GFP) by its characteristic fluorescence intensity at 509 nm, while a standard dye simultaneously reported on the temperature of the solution. Each snapshot experiment simultaneously measured the extent of denaturation from 44 to 85°C, using a single 15.3 nL sample, and just 2.6 seconds for image acquisition at each time-point, allowing the time and temperature-dependence of protein denaturation to be readily obtained. Analysis of a complete thermal denaturation profile (24 to 85°C), from a single time-point at 30 seconds after heating, gave accurate thermodynamic parameters in agreement with those obtained from a conventional water-bath controlled fluorimeter experiment taking 170 minutes with a 0.2 mL sample.

## Results

**Figure 1:**
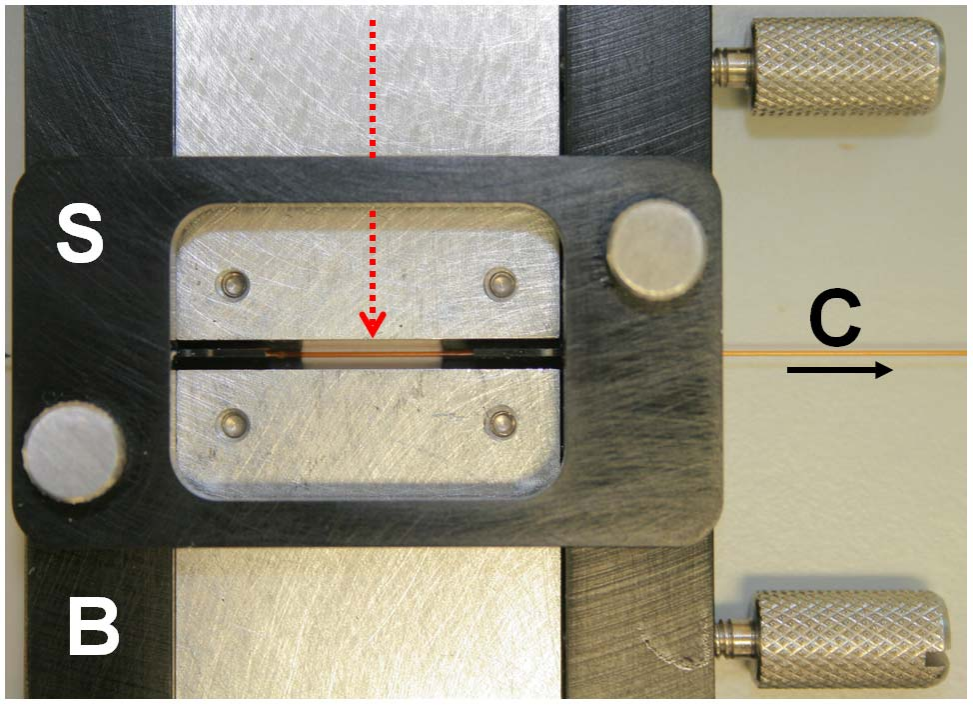
A custom-made butterfly stage (B) aligns an IR laser via embedded optical fibre (red arrow) to a micro-capillary (C, arrow shows flow direction) clamped with a setting tool (S).

**Figure 2:**
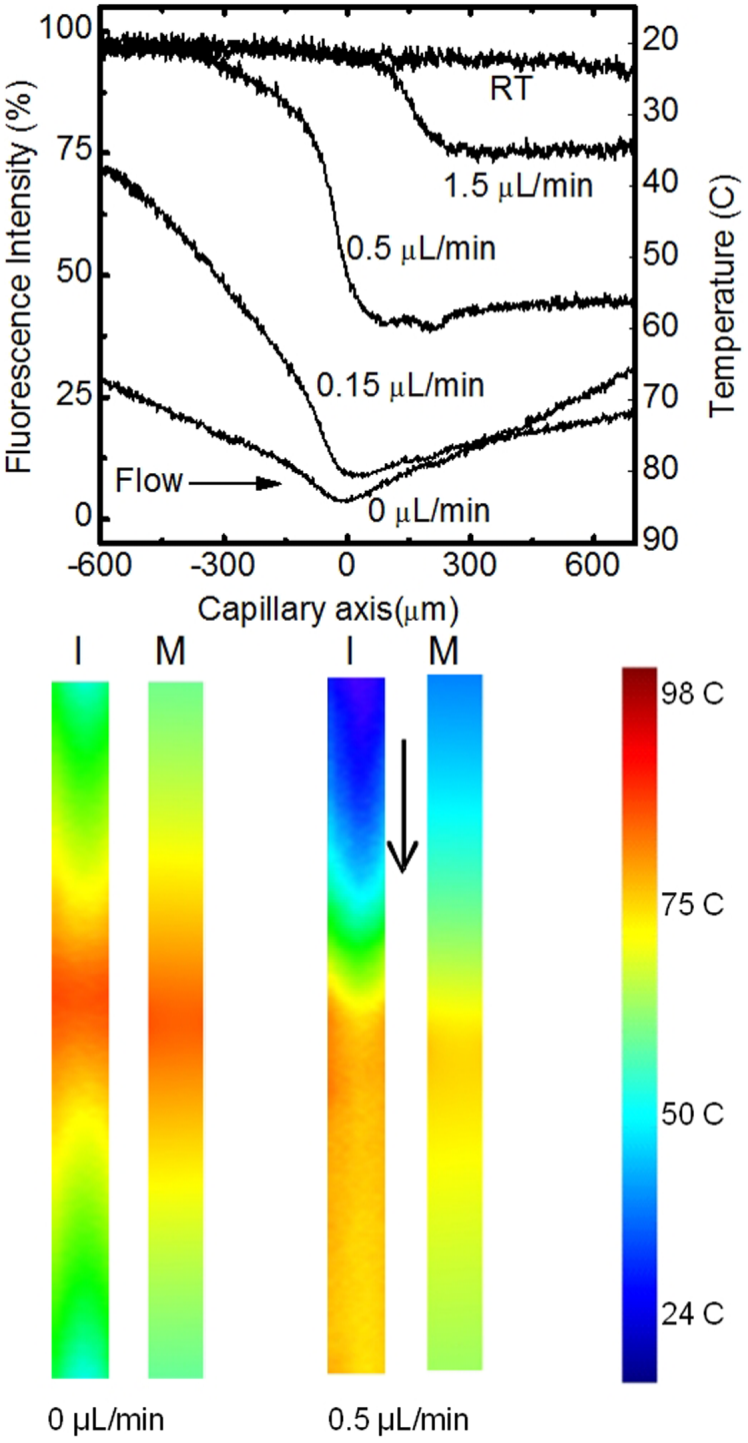
Top: Relative FLI of TAMRA at various flow rates and fixed laser power. RT denotes room temperature (no laser). The 5 μm diameter laser spot was located at zero on the capillary axis. Bottom: Confocal images (I) and COMSOL model (M) data at zero flow and 0.5 μL/min in the direction of arrow.

To initially characterise optical point source heating of proteins in microfluidic channels, we used confocal microscopy, and a stage of our own design that precisely aligned an IR-laser (wavelength = 1480 nm) beam perpendicular to capillary flow via an embedded fibre optic (Fig. 1). The first step in the experiments was to image the temperature distribution in three dimensions at micron resolution, as a function of time after switching on the IR laser, laser power and flow rate. This was achieved by monitoring the emission from the temperature-sensitive fluorescent dye tetra-methylrhodamine (TAMRA)^20^, calibrated with a conventional fluorimeter (Fig. S1, Supporting Information), heated using a ‘pump' laser aligned perpendicular to the capillary axis. In Fig. 2 we show average FLI values for TAMRA, and corresponding temperatures, at each point along the capillary length (X-axis), obtained from all Y-axis values in a single XY-plane at the capillary Z-axis centre, at 150 mW IR laser power (measured at the output of the embedded fiber), and flow rates from 0–1.5 μL/min, in comparison to an unheated (room temperature) sample. The sample flow direction is indicated by the horizontal arrow. The FLI distribution over the whole XY plane is shown in Fig. S2A and Fig. S2B (Supporting Information). At zero flow the temperature distribution is symmetrical, with a minimum fluorescence corresponding to 85°C, at the centre of the capillary where the IR laser spot was aligned. The temperature change of ≈65°C was instantaneous relative to the image acquisition time (2.6 s) of the confocal microscope, as observed by eye. Increasing the flow rate (left to right), at constant laser power, changed the shape of the FLI curves where the point of maximum temperature (*T*_max_) shifted downstream from the point of heating, while *T*_max_ also decreased. At higher flow rates of 0.5 μL/min and 1.5 μL/min, the curve assumed a step function whereby the temperature downstream of the point of heating remained almost constant along the capillary length.

The flow experiments allowed us to resolve heating rates for water in our system. At f = 0.5 μL/min, implying a flow velocity v = f/(πr^2^) ≈ 740 μm/sec, the temperature increased by 40°C over a total flow distance of 300 μm, corresponding to ≈400 ms. The peak rate of change was 0.23°C/ms. Similarly, at 1.5 μL/min, the temperature increased by 15°C over 150 μm, with a peak of 0.22°C/ms. After scaling laser powers and taking into account fiber and coupler losses in our system, the peak rate of change of temperature of 0.22°C/ms is of the correct order when we consider that Braun and co-workers obtained 7°C/ms using a 1.2W IR laser with free space optics to heat a static DNA sample between two microscope slides^20^.

The IR laser-induced heating of a liquid under microfluidic capillary flow was modelled in COMSOL Multiphysics by combining the inbuilt heat-transfer and fluid dynamic modules. Experimental confocal images of TAMRA fluorescence converted into temperature maps compare well to corresponding models in COMSOL (Fig. 2; bottom panel). Experimental temperature values were reproduced to within a few degrees Celsius, correctly predicting the effect of flow on the temperature distribution. The model therefore shows that heat transport occurs through both diffusion and advection to create the observed temperature distributions. The agreement with experiment also indicates its use for engineering future integrated optical/microfluidic chips, where the model can determine combinations of mass-flow and optical heating to achieve desired time and space-dependent thermal profiles.

### Protein stability measurements

Having established thermal gradient control, we demonstrated the rapid measurement of protein stability to thermal denaturation using the well characterised green fluorescent protein (GFP), whose intrinsic fluorescence conveniently reports upon its folded state^21^,^22^,^23^,^24^.

The absorption spectrum of recombinant turbo GFP (rTurbo-GFP) peaks at around 470 nm, and upon denaturation the fluorescence is quenched by the solvent. The unfolding of GFP due to optical-heating in a microcapillary was imaged as a fluorescence intensity decrease using the same stage and confocal microscope as for the TAMRA experiments. The concentration dependence of denaturation at 0.001 mg/ml to 0.05 mg/ml GFP and 100 mW laser power was found to give 2–10% of the fluorescence intensity relative to that at 24°C, with no clear trend (Fig. S3, Supporting Information). However, the fluorescence intensity of GFP denaturation showed a time-dependence below 30 seconds at 0.001 mg/ml (Fig. S4, Supplementary Information), and typically 1–20 minutes at the higher concentrations (data not shown). Furthermore, an experiment in which stepwise increases in laser power, followed by stepwise decreases, resulted in up to 50% loss in the recovery of the initial GFP fluorescence intensity (Fig. S5, Supporting Information). Together these experiments indicated that GFP denaturation was partially irreversible, due to protein aggregation, irreversible misfolding, or capillary fouling in which proteins adhere to the capillary inner walls. All subsequent experiments were carried out at the lowest GFP concentration of 0.001 mg/ml, while retaining a cleaning protocol between tests as previously described^8^.

**Figure 3:**
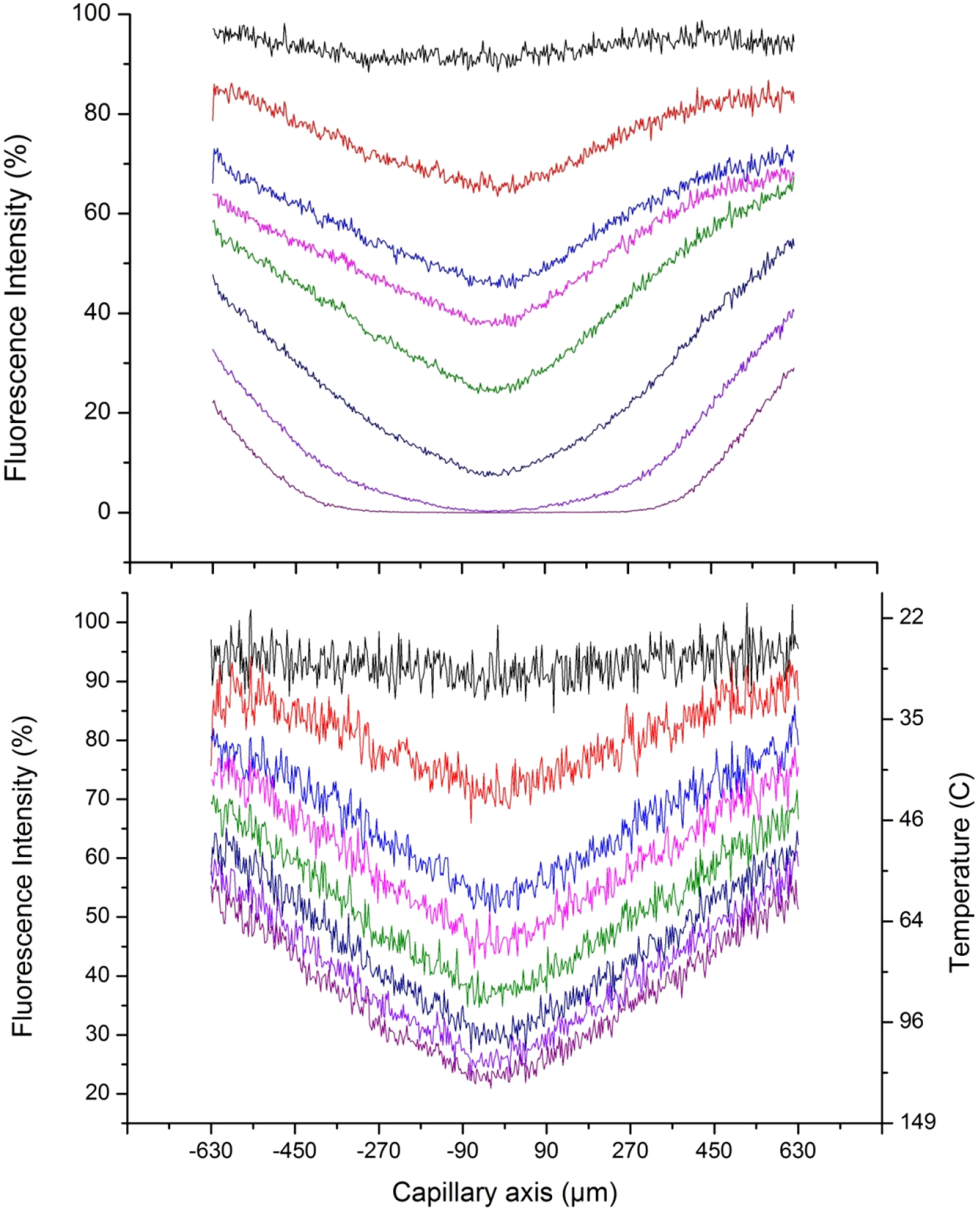
Fluorescence intensities relative to those at 24°C for GFP (top panel), and TAMRA (bottom panel with corresponding temperatures), for 25 mW (black) to 150 mW (dark purple) IR laser power.

Various temperature profiles, at zero flow, were achieved using the IR laser at a range of input powers using either TAMRA and 0.001 mg/ml GFP separately (data not shown), or with both samples combined and using multicolour imaging, to confirm that TAMRA did not alter the temperature dependence of GFP fluorescence. The optically induced heating of TAMRA and GFP combined in a single sample enabled more convenient and precise determination of GFP denaturation curves by circumventing variations in laser power, or errors due to different mechanical alignments of the capillary. Figure 3 shows typical data for eight laser powers increasing from 0–150 mW. Confocal images, acquired in 2.6 seconds for high resolution, were obtained before and after IR-induced heating commenced by scanning length-wise along the capillary axis. The confocal images were acquired every 30 seconds, typically for up to 5–20 minutes. Each image was then analysed to give a temperature profile of GFP fluorescence as a function of time (Fig. S4, Supporting Information). No time-dependence was observed for TAMRA fluorescence with this method, nor at a lower resolution image acquisition of <1 second, indicating that the IR-induced response of TAMRA was faster than that timescale.

**Figure 4:**
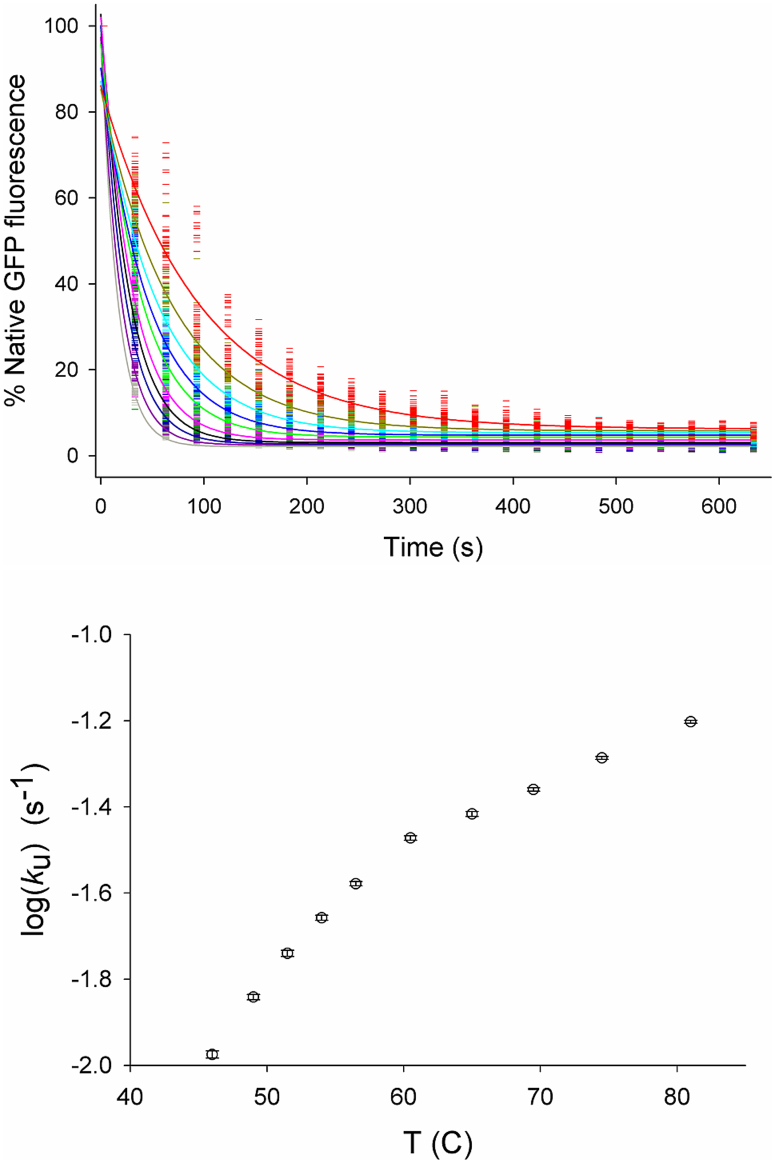
Top: GFP denaturation decay curves at selected temperatures from 46–81°C obtained by fluorescence image scanning at 30 s intervals for a single IR-heated capillary region. Lines were best fit to equation Eq. 1, and shown at 46°C (red), 49°C (dark yellow), 51.5°C (cyan), 54°C (blue), 56.5°C (green), 60.5°C (pink), 65°C (dark green), 69.5°C (dark blue), 74.5°C (dark pink), 81°C (grey). Bottom: Temperature dependence of the logarithm of the rate constant, log(*k*_u_), of GFP denaturation, obtained using the optical heating method, and fitting decay data to Eq. 1.

The FLI for TAMRA and GFP both decreased as the laser power, and temperature were increased, and their profiles remained symmetrical at all laser powers. The TAMRA fluorescence was used to directly determine the temperatures associated with the GFP fluorescence intensity at the same location in the capillary. Time-dependent GFP fluorescence decay curves were obtained using these data, over the range 44 to 85°C (Fig. 4), and fit to Eq. 1 to give the rate constant, *k*, for GFP denaturation as a function of temperature (Fig. 4), for a single 15.3 nL sample. The initial GFP fluorescence intensities (Fig. 4, top panel) for various temperatures are all normalized to 100%. At lower temperatures, the rate of decrease of FLI is slower than at higher temperatures.

The logarithm of the unfolding rate constant, log(*k*_u_), obtained (Fig. 4, bottom panel) was non-linearly dependent upon temperature, with a kink at approximately 60°C, indicating a mechanistic change at that temperature. This is likely to be due to the shift from reversible to irreversible unfolding at 60°C. Our unfolding rate at 70°C, pH 7.5, as well as the loss of complete recovery upon re-cooling is in excellent agreement with previous work at pH 7.2 using conventional water-bath control^25^.

**Figure 5:**
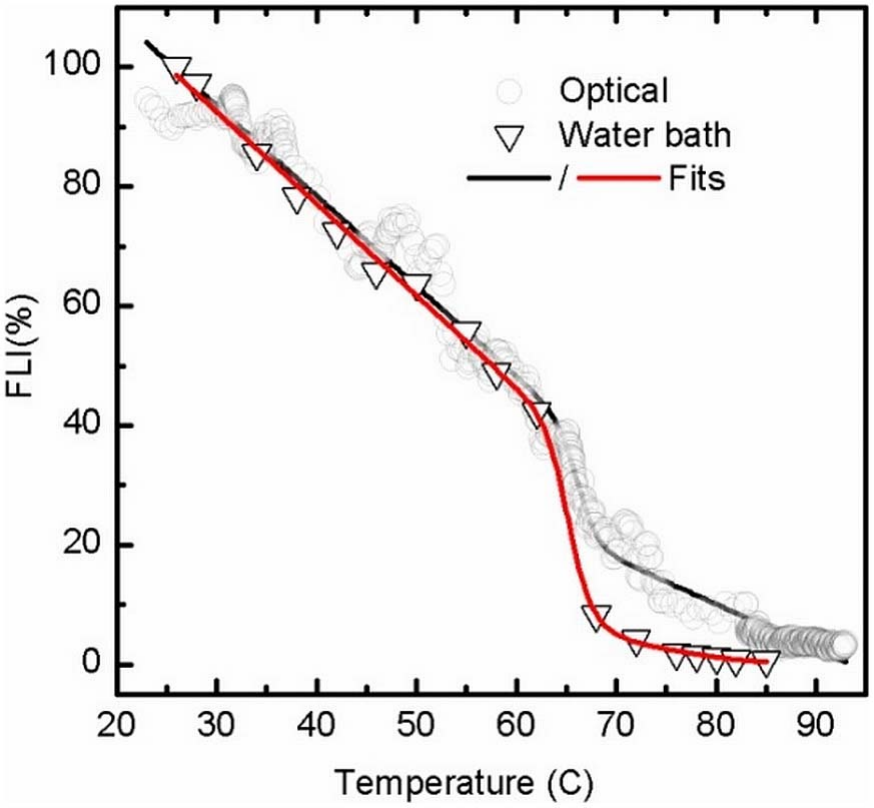
Thermal denaturation of GFP fluorescence, using optical and conventional water-bath heating methods. Data are fit to Eqs. 2 and 3.

In an alternative experiment, data from three confocal images acquired after 30 seconds of heating at different laser powers, but reaching temperatures spanning overlapping ranges, were combined to obtain a single complete thermal denaturation profile for GFP (Fig. 5). The transition region itself (60–70°C) was obtainable within a single scan covering 44 to 85°C from just one laser power. The thermal dependence of TAMRA and GFP FLI obtained was compared to that acquired with a conventional water-bath controlled fluorimeter. The conventionally obtained denaturation profile took 0.2 ml of sample and a total of 170 mins, due to the equilibration of the water bath and sample requiring 10 mins between each 2°C or 4°C step. By contrast, the microfluidic method measured a 15.3 nL sample and acquired the whole thermal denaturation curve after a single 30 seconds incubation, without requiring sequential step-wise temperature increases of the sample.

The two denaturation curves in Figure 5 were fit to a standard equilibrium two-state thermal denaturation equation (despite observing up to 50% loss of reversibility above the transition region), to give indicative thermal transition midpoint temperatures, *T*_m_, of 66.3 ± 0.1°C and 65.5 ± 0.9°C, respectively for the optical and water bath heating methods. These matched to within 0.5°C and the two unfolding curves were in excellent agreement. The degree of fluorescence quenching above the *T*_m_ was slightly lower for the optical heating experiment. This is due to the significantly shorter total incubation time, and thus greater protection from slow irreversible misfolding, aggregation or fouling processes.

This demonstrates another potential advantage of the technique over standard water-bath based instruments, where reversible unfolding stability and kinetic measurements could now be obtained rapidly for certain proteins, such that slower irreversible aggregation does not yet predominate.

## Discussion

In this study we have developed a novel ‘all-optical' technique that combines optically-induced ‘point of contact' heating, controlled mass-flow with nanoliter volume samples and computational fluid dynamics to account quantitatively for the experimental phenomenology. The observed systematic variation in temperature (Fig. 2) of the test sample TAMRA with flow rate is modelled using COMSOL to unprecedented accuracy and is further supported by back of envelope calculations (Supplementary Information). Upon the successful understanding of the physics behind these experiments, we have applied this technique to demonstrate denaturation of a prototypical protein –GFP. The thermal denaturation temperature *T*_m_ obtained using this technique is in agreement with the literature. Another significant application of this technique is the measurement of rate constants of protein denaturation (Fig. 4) at various temperatures.

Further, our technique shows for the first time that it is possible to obtain protein stability parameters, and the temperature dependence of unfolding kinetics, by optically induced heating of nanolitre samples in a single snapshot experiment. Coupled to microfluidic detection methods such as for the intrinsic fluorescence of proteins that we demonstrated previously^8^, the well-controlled, directional, and local heating achievable makes this a promising tool to examine a broad range of temperature-dependent biological and chemical processes on millisecond to minute timescales, in nanolitre sample volumes, as well as for point of care, diagnostic assays including those entailing qPCR^19^.

## Methods

All samples were obtained from Sigma (SIAL Ltd, Poole, UK) unless otherwise stated.

### Calibration of GFP and TAMRA temperature dependence

The temperature dependent fluorescence intensities of 0.001 mg/ml GFP (Biocat GmbH, Heidelberg, Germany) and 0.025 mg/ml TAMRA (Invitrogen Corp., Carlsbad, CA) were determined with a Fluoromax-3 fluorescence spectrometer (Horiba Jobin Yvon Ltd, Stanmore, UK), for 0.2 mL samples in a 1 mm path-length quartz cuvette with temperature increased using a water-bath from 24°C to 85°C ± 1°C in 2°C or 4°C steps every 10 minutes, to allow equilibration. Fluorescence intensity of TAMRA at each temperature, relative to that at 24°C, enabled us to determine the temperature of IR-laser heated samples in a capillary from the equivalent relative TAMRA fluorescence intensity.

### Confocal imaging of IR-heated samples in a micro-capillary

The plastic coating of a fused-silica glass micro-capillary (FS-110, Upchurch Scientific, ID = 120 μm, OD = 360 μm) was removed with a blue flame and the capillary clamped into a custom-made confocal microscope butterfly stage (RoMack Inc., Williamsburg, VA) indicated as *B* in Fig. 1. The stage provided unobscured access to the top and bottom of the capillary for confocal microscopy over a 10 mm length containing a sample volume of 113 nL. It also accurately positioned a micromachined aluminium block containing an embedded single-mode optical fibre, to align the polished fibre ends perpendicular to the capillary length (X-axis) at the centre of the visible length. A FITEL 1480 nm IR-laser (Furukawa Electric Europe Ltd., London, UK) was standard FC/PC coupled to the optical fibre to provide localized heating of samples within the capillary with a 5 μm diameter laser spot at up to 180 mW.

Liquid samples were pumped into the micro-capillary at 0–1.5 μL min^−1^ using a 100 μL Hamilton gas-tight syringe and syringe pump (KD Scientific Inc., Holliston, MA). For measurements at 0 μL min^−1^, the open end of the micro-capillary was sealed with soft rubber to minimize the movement of the liquid inside the tube. The fluorescence intensity of 0.025 mg/ml TAMRA in 18 MΩ.cm ultrapure water was imaged with 543 nm laser excitation and fluorescence emission at 590 nm with a 100 nm transmission window, by scanning length-wise along the capillary axis on a laser scanning confocal microscope (Fluoview-FV 1000, Olympus). The temperature distribution of aqueous samples was mapped over 15 planes at 5 μm Z-axis steps within the capillary. Each 2D plane image was obtained for an X and Y axis lateral step-size of 1.38 microns. The capillary sample was heated at a range (0–150 mW) of IR laser powers to generate different temperature profiles, and confocal images obtained in 2.6 seconds, after a 5 second pause, by scanning length-wise along the capillary axis. Each 2D plane image of the scanned capillary, taken at different Z-axis positions, was analyzed using ImageJ software^26^ to obtain the FLI values at each point on the image plane.

### IR-induced thermal denaturation of nanolitre GFP samples

For thermal denaturation, turbo-GFP was prepared at 0.001, 0.02 or 0.05 mg/ml in 50 mM Tris. HCl, pH 7.2, both in the presence and absence of 0.025 mg/ml TAMRA*.* GFP fluorescence was imaged as for TAMRA above, but with 488 nm laser excitation and fluorescence emission at 520 nm with a 100 nm transmission window. Thermal denaturation curves for GFP were obtained in two parallel ways in a micro-capillary. In one, fluorescence images were obtained for separate GFP and TAMRA samples using the same set of IR-laser powers, and the two datasets aligned afterwards. The second method eliminated potential systematic errors by using multi-color fluorescence imaging on samples containing both TAMRA and GFP. Two lasers provided excitation of TAMRA and GFP and images of their respective fluorescence emissions obtained sequentially. Image scans, taking 2.6 seconds, were repeated every 30 seconds to obtain time-resolved data. The GFP fluorescence intensity data and spatially corresponding TAMRA fluorescence data for each time-point were converted to temperature, and pooled from scans performed at up to seven IR laser powers from 0 mW to 150 mW, and XY-plane images taken at a Z-axis depth corresponding to the capillary centre. The GFP FLI relative to the value at 24°C was then plotted against the corresponding TAMRA-derived temperature values for a given time point, or otherwise plotted as GFP FLI as a function of time at each temperature.

For time-dependent GFP FLI, the rate constant for denaturation were obtained by fitting the data to equation S1 in Sigmaplot (Systat Software Inc, Chicago, IL, USA), as shown in Figure S6. 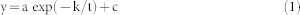
where a is the signal amplitude, c is the fluorescence signal decay endpoint, and k is the rate constant for the fluorescence signal decay associated with GFP denaturation.

The thermal denaturation profiles for GFP at a given time-point were fit in Sigmaplot to a two-state folding model, using Equations 2 and 3 combined. 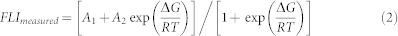

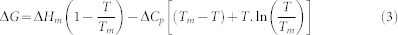
where *A*_1_ and *A*_2_ are linear pre- and post-transition baseline functions, *R* is the universal gas constant, and Δ*C*_p_ = 2856 cal/mol/deg assuming Δ*C*_p_ = 12 cal/mol/deg/residue^27^,^28^.

### CFD model of IR-induced fluid heating in a microcapillary

The laser-induced optical heating in the microfluidic capillary was modeled using COMSOL v3.5 (COMSOL Inc., Burlington, MA) by combining the inbuilt heat-transfer and fluid dynamic modules, with the experimentally determined laser power first modeled as a heat source with power decaying as per an extinction coefficient of 21.2 cm^−1^ at 1480 nm over the 120 μm inside capillary diameter. The weakly compressible Navier-Stokes equation for non-isothermal fluid flow (Eq. 4) was combined with the standard heat-diffusion equation^31^ (Eq. 5), assuming non-isothermal flow: 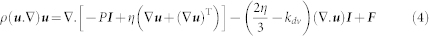

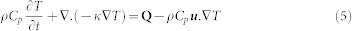
where, *ρ* is the density of water, *C_p_* is the specific heat, *T* is temperature, *Q* is the heat source, ***u*** is the velocity of flow, ***I*** denotes a unit vector, *η* is the dynamic viscosity, *k_dv_* is the dilational viscosity, and ***F*** is the volume force.

The coupled equations were solved simultaneously in COMSOL using parameter values provided in the standard COMSOL library, for density and viscosity of water, etc., unless otherwise stated below. The heat transfer coefficient h = 10 W/m^2^.K (water to glass), surface emissivity, e = 0.8 (glass surface to ambient air), T_in_ and T_amb_ = 24°C (ambient and initial temperature), the inner-diameter of the capillary = 120 microns, and the Radiation type ‘surface-to-ambient' switch was on. A rough estimate of heat-diffusion time of 30 ms can be obtained by^29^, 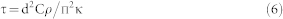
where d is the capillary wall thickness, C is the heat capacity of water, *ρ* the density, and κ the thermal conductivity (0.54 W/K m). This is much faster than the image acquisition timescale, thus ensuring thermal equilibrium. At typical flow rates used (0.5 μL/min), the flow velocity of 7 × 10^−4^ m/s is much greater than the maximum thermal convection velocity of 1.4 μm/s^30^. Thus, our observations are not affected by thermophoresis. Further, we believe that the possible influence of thermophoresis on our protein denaturation experiments is either minimal or nil due to the following reasons: a) As the temperature increased from 25 to 60°C the GFP fluorescence intensity in the cooler regions did not increase as would be expected if the GFP concentration increases by movement into the cooler regions; b) the slope of FLI vs T over 25 to 60°C (Fig. 5) is exactly coincident with that from the water bath method for which thermophoresis is not relevant; c) the GFP unfolding rate constant measured in this study (Fig. 4) matches previous bulk equilibrium measurements at pH7 and 70°C (Alkaabi *et al*. *2005*). However, we cannot completely discard the effect of temperature-dependent diffusion on thermophoresis.

## Author Contributions

The manuscript was written through contributions of all authors.

## Supplementary Material

Supplementary Information Supplementary Information
