# Supplementary material for: Optically Induced Thermal Gradients for Protein Characterization in Nanolitre-scale Samples in Microfluidic Devices
Source: Sci Rep. 2013 Jul 4;3:2130. doi: 10.1038/srep02130 (PMC3703920; doi:10.1038/srep02130)
Supplement: Supplementary Information [file srep02130-s1.pdf]

Supplementary Information for: Optically Induced Thermal Gradients for Protein Characterization in Nanolitre-scale Samples In Microfluidic Devices

D. M. Sagar[†,‡], Samir Aoudjane[†,‡], Matthieu Gaudet[†,‡], Gabriel Aeppli[‡] and Paul A. Dalby[†*]

[†] Department of Biochemical Engineering, Torrington Place, University College London, London, WC1E 7JE, U.K

[‡] London Centre for Nanotechnology and Department of Physics and Astronomy, University College London, London, WC1H 0AH, U.K

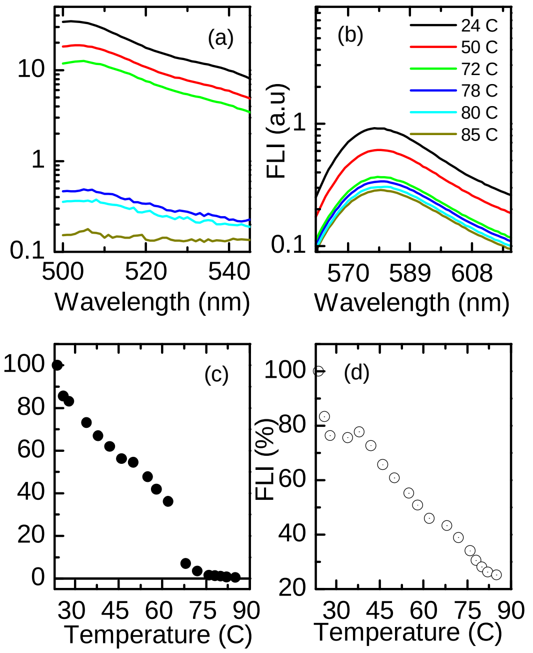

Figure S1. Calibration of the temperature-dependent fluorescence of GFP and TAMRA. Fluorescence spectra for 0.001 mg/ml GFP (a) and 0.025 mg/ml TAMRA (b) were obtained at various temperatures set using a water bath on a fluorescence spectrometer. Temperature dependent FLI values were obtained for GFP at 520 nm (c), and TAMRA at 580 nm (d) where FLI values were determined as the %FLI relative to that at 24 °C (100%) in both cases.

In both Fig. S2a and S2b, and also at room temperature (not shown) the fluorescence intensity distribution of TAMRA peaks at the centre of the radial axis (X-axis) of the capillary. This distribution reflects the radial symmetry of the capillary with the intensity weight concentrated around the centre and decreasing significantly towards the capillary walls perpendicular to the incident direction of confocal imaging lasers. Qualitatively this results from multiple compounded effects, including a cylindrical lensing effect caused by the capillary glass, optical aberrations due to possible astigmatism, as well as phase-space filling arguments. As the scanning laser traverses across the radial dimension of the capillary, the probed volume scales with the available phase-space in the capillary. Thus, at the centre, the available phase-space is at its maximum, while at the peripheries it is at the minimum. The phenomenon is identical when no heat is applied and therefore cannot be due to the formation of a thermal gradient perpendicular to the capillary axis. A more detailed analysis of all these phenomena is beyond the scope of the present work. As can be seen in each case, profiles are the same at each X-axis position, and so the phase space effect does not impact the average signal as a function of distance along the capillary length, as was used to obtain thermal denaturation profiles. In all analyses the central 3/4 of the radial width was used for quantitation which minimised any possible aberrations due to non-paraxial rays causing a cylindrical lensing effect.

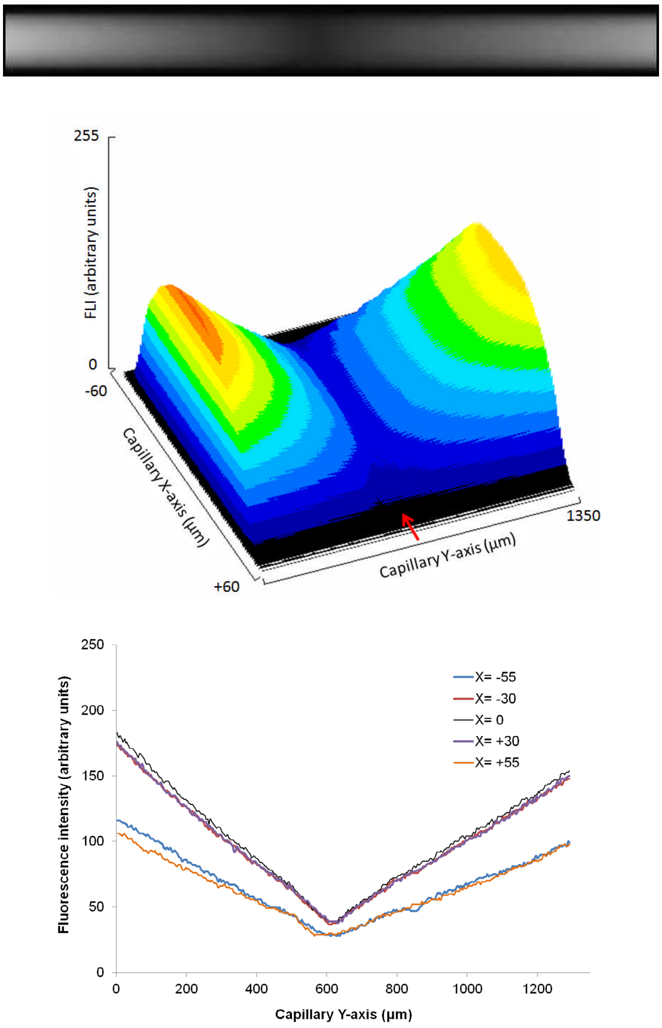

Figure S2a. FLI distribution over the XY plane image at the central Z-axis depth in the microcapillary, with no sample flow. Upper image is obtained from the confocal microscope, which is then converted into a 3D surface plot of fluorescence intensity (Middle image). The arrow marks the position of the I.R. laser spot. Lower image shows the profiles of fluorescence intensity as a function of capillary Y-axis, at 5 different capillary X-axis positions.

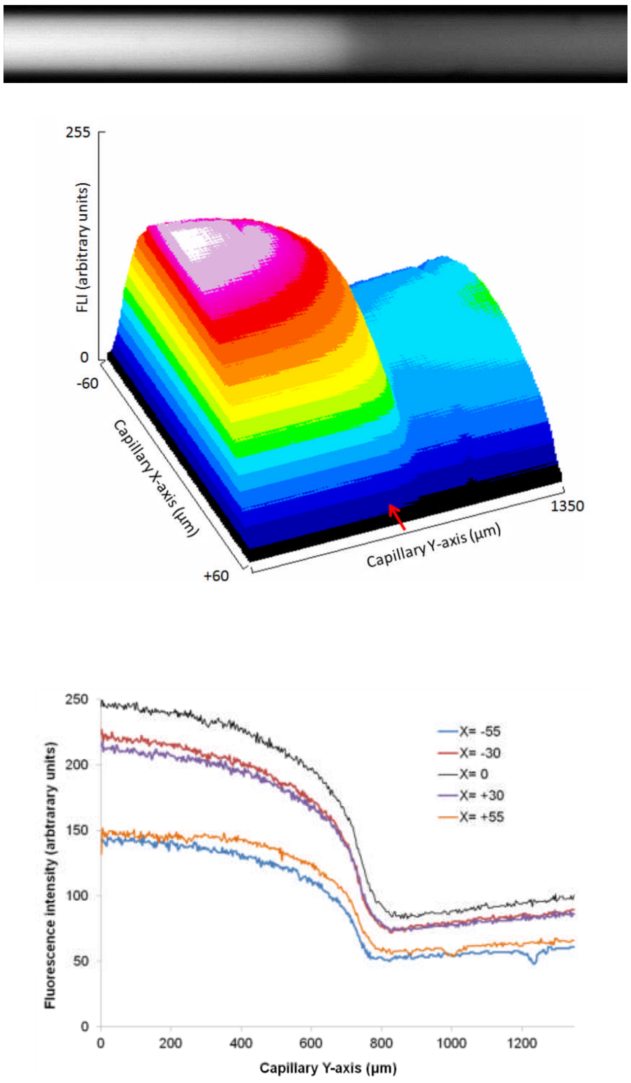

Figure S2b. FLI distribution over the XY plane image at the central Z-axis depth in the microcapillary, with sample flow at 0.5 µl/min. Upper image is obtained from the confocal microscope, which is then converted into a 3D surface plot of fluorescence intensity (Middle image). The arrow marks the position of the I.R. laser spot. Lower image shows the profiles of fluorescence intensity as a function of capillary Y-axis, at 5 different capillary X-axis positions.

Figure S3. GFP concentration-dependence of fluorescence intensity expressed as % of that at 24 °C in the microcapillary, using 100 mW laser power (for a temperature of approx. 75 °C). No systematic variation of FLI values was observed for a given laser power. Therefore the protein unfolding was

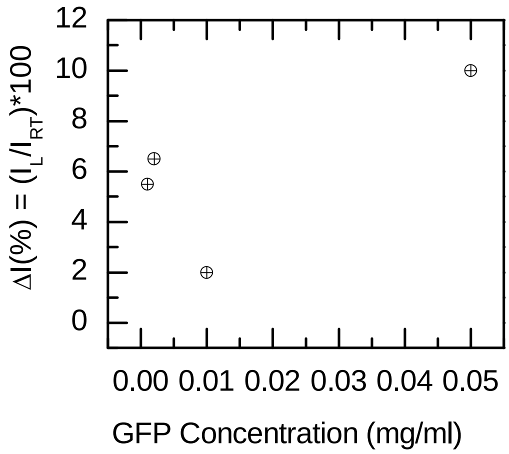

independent of protein concentration. All these experiments were performed consecutively in order to avoid possible experimental errors.

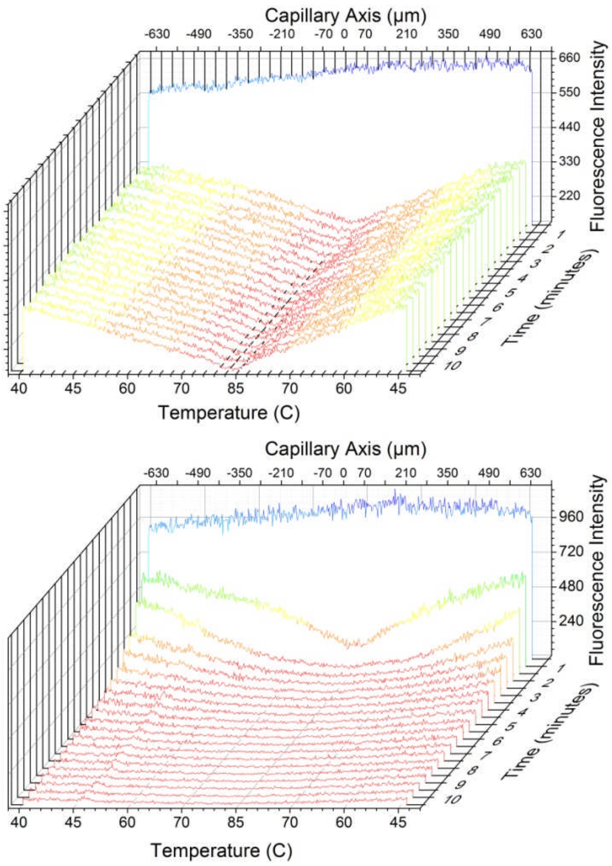

Figure S4. Typical time-dependent FLI traces of TAMRA (top) and 0.001 mg/ml GFP (bottom) after switching on the IR laser (in this case to a maximum temperature of 85 °C). Each trace corresponds to 30 seconds. Zero microns correspond to the position of the IR laser.

Fig. S4 shows the time-dependence of fluorescence intensity for both TAMRA and GFP after inducing heating with the IR laser. The traces were recorded every 30 seconds at a fixed laser power for 10 minutes. As seen, no measurable time dependence in FLI from TAMRA is observed over the course of the experiment, whereas 0.001 mg/ml GFP demonstrates a time-dependent decay related to the rate of thermal denaturation of GFP. Even longer time dependences were observed at higher protein concentrations (data not shown), indicating an increased occurrence of protein aggregation or capillary fouling.

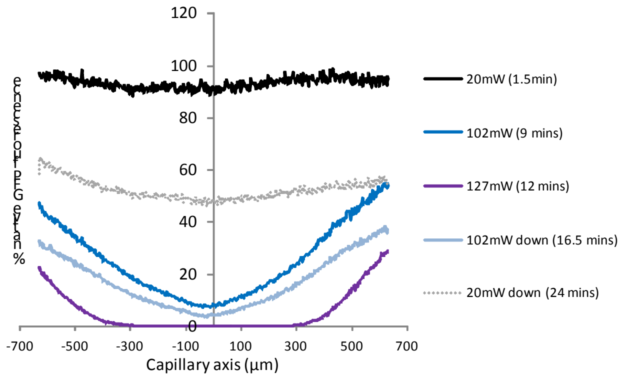

Figure S5. Incomplete recovery of GFP quenching after stepwise re-cooling. The IR power was stepped up every 1.5minutes (20mW, 38mW, 58mW, 68mW, 85mW, 102mW, 115mW, 127mW), and then back down again for comparison. Times in brackets indicate the time-point for the total experiment using a single sample. The fluorescence intensity of 0.001 mg/ml GFP was found not to recover completely, indicating a partially irreversible unfolding process. The TAMRA fluorescence was fully reversible, indicating that the temperature had equilibrated at each step during both heating and cooling.
